# Incidence of optic nerve kinking in a cohort of patients with Normal tension glaucoma

**DOI:** 10.1038/s41433-025-03608-5

**Published:** 2025-01-18

**Authors:** Achmed Pircher, Jatta Berberat, Luca Remonda, Cynthia J. Roberts, Albert Neutzner, Hanspeter E. Killer

**Affiliations:** 1 Department of Neuroscience/Ophthalmology, Uppsala University, Uppsala, Sweden; 2 Cantonal Hospital, Institute of Neuroradiology, Aarau, AG Switzerland; 3 Departments of Ophthalmology & Visual Sciences; and Biomedical Engineering, The Ohio State University, Columbus, Ohio, USA; 4 Department of Biomedicine, University of Basel, Basel, Switzerland

**Keywords:** Medical research, Outcomes research

## Abstract

**Objectives:**

To report on the incidence of optic nerve kinking in a series of patients diagnosed with normal-tension glaucoma (NTG) compared to an age- and gender matched control group without known optic nerve diseases.

**Subjects and Methods:**

All patients with NTG who underwent imaging (computed tomography cysternography (CTC) or magnetic resonance imaging **(**MRI)) of the orbits and cranium between 2012 and 2022 were included, totalling 57 patients (27 females and 30 males; 57 eyes; mean age 69 ± 10 years). 57 age- and gender matched subjects without known optic nerve diseases who underwent MRI of the orbits and cranium served as controls. Radiographic images of the orbits were analysed for the presence of optic nerve kinking.

**Results:**

In the axial plane at least one optic nerve kink was found in 49 of 57 (86%) optic nerves in patients with NTG and in 10 of 57 (18%) optic nerves in controls (*p* < 0.0001) while in the sagittal plane in 28 of 57 (49%) optic nerves in patients with NTG and in 1 of 57 (2%) optic nerves in controls (*p* < 0.0001) (Fisher’s two-tailed exact test).

**Conclusions:**

This study demonstrates a high statistically significant incidence of optic nerve kinking in patients with NTG compared to controls without known optic nerve diseases. Its possible role involved in the pathophysiology of NTG needs to be evaluated.

## Introduction

Normal tension glaucoma (NTG), a variant of primary open angle glaucoma (POAG), is a chronic progressive optic neuropathy that is characterized by visual field loss and optic disc cupping but with normal intraocular pressure (IOP). [1] IOP lowering can result in slowing the disease progression, although there is a substantial number of patients that progress despite successfully lowered IOP [2]. IOP independent risk factors must therefore be considered in the pathophysiology of NTG, among them vascular dysregulation, [3] the translaminar pressure gradient, [4] mechanical impact of the lamina cribrosa [5] and neurodegenerative mechanisms. A possible correlation between NTG and Alzheimer’s disease is suggestive for the role of a cerebrospinal fluid (CSF) in the pathophysiology of NTG [6].

In contrast to other cranial nerves, the optic nerve is a white matter tract of the brain that is surrounded by cerebrospinal fluid within the subarachnoid space over its entire length from intracranially (pre-chiasmatic portion) up to the lamina cribrosa (bulbar portion).

CSF enters the subarachnoid space of the optic nerve from the suprasellar cistern and flows through the optic canal into the intraorbital portion of the subarachnoid space towards the lamina cribrosa that ends in a cul de sac fashion. The outflow route of CSF from the optic nerve subarachnoid space is not yet fully understood, but it is conceivable that lymphatic clefts in the dura of the optic nerve might provide an outflow route [7, 8].

Next to its mechanical function as a buffer to protect the brain and the optic nerve from mechanical injuries, CSF acts as a transport system for the supply of nutrients and neurotransmitters to neurons and axons. On the other hand, it functions as a “sewage system” that removes toxic biological debris from the brain in combination with the glymphatic system. The integrity of a continuous CSF in- and outflow along the optic nerve is therefore of great importance. Disturbances of cerebrospinal fluid dynamics result in neurodegenerative disease, like Alzheimer’s dementia, in which accumulation of amyloid beta protein leads to plaque formation [9].

It has been demonstrated in patients with NTG that the CSF flow along the optic nerve can become impaired due to compartmentalization of the optic nerve sheaths. [10] This process can lead to localized pressure gradients and stagnation of CSF dynamics within the optic nerve subarachnoid space, with consecutive accumulation of potentially neurotoxic substances [11].

Optic nerve tortuosity is the radiographical description of an abnormally curved optic nerve which in a more pronounced extent (larger angle) is described as “optic nerve kinking”. In the current study we prefer to use the term “optic nerve kinking” to “optic nerve tortuosity” because we examined the orbital optic nerve for the presence of larger angle kinks. Optic nerve kinking has been found in about 30–40% of patients with idiopathic intracranial hypertension (IIH) [12]. It is also described in association with papilledema in patients with central sinus venous thrombosis, [13] cases with neurofibromatosis [14] and Chiari malformation [15]. The most recently described cases of optic nerve tortuosity were found in astronauts with space flight associated neuro ocular syndrome (SANS) [16]. Elevated intracranial pressure seems the common denominator behind most of the cases of optic nerve tortuosity.

This study systematically examines computed tomography cisternography (CTC) and magnetic resonance imaging (MRI) images of the orbits and cranium in 57 NTG patients and 57 age- and gender-matched controls without known optic nerve diseases for the presence of optic nerve kinking in the axial and sagittal planes. The study was undertaken to investigate about possible anatomical features of the optic nerve that might influence the CSF flow dynamics in the subarachnoid space along the optic nerve in patients with NTG.

## Subjects and Methods

The study was approved by the local ethical commission and follows the tenets of the Declaration of Helsinki. A signed informed general consent for the use of patient’s data is available for all patients and controls included in the study.

This study is a retrospective case-control study that compares the incidence of optic nerve kinking in NTG patients with controls without known optic nerve diseases.

### Normal tension glaucoma Patients

Medical records of patients with NTG from 2012 to 2022 of the Department of Ophthalmology at our tertiary referral hospital were analysed. All patients that fulfilled the inclusion criteria for NTG that underwent imaging of the orbits and cranium (CTC or MRI) were included.

NTG was diagnosed based on glaucomatous optic disc cupping on ophthalmoscopy and concomitant visual field defects. Intraocular pressure (IOP) maximum using Goldman applanation tonometry was always found to be < 21 mmHg and the visual field mean deviation (MD) was ≥ 3 decibel (dB) documented by using standard automated perimetry (Program G2, Octopus Haag-Streit, Switzerland) at time of imaging. Each patient underwent several full ophthalmologic examinations including slit lamp assisted biomicroscopy, applanation tonometry, gonioscopy, ultrasound pachymetry, perimetry and neuroretinal rim assessment by optic coherence tomography of the optic nerve head (Heidelberg engineering, California, USA). In each patient IOP and visual field was measured regularly over a period of at least three years. In all patients, IOP was measured at least once at different times during 24 h (between 8 a.m. and 8 p.m.) in a seated position, applying Goldmann applanation tonometry and twice at night (between 9 p.m. and 6 a.m.) in a supine position, using Perkins tonometry by an ophthalmologist during a 24 h IOP screening in the hospital. IOP lowering treatment consisted of topical applied carbonic anhydrase, b-blockers, prostaglandin analogues, a-agonists, and combinations of these medications.

### Controls

From the database of the Department of Neuroradiology at our tertiary hospital, 57 age- and gender-matched individuals who underwent MRI of the orbits and cranium were recruited in a retrospective manner and served as controls. All patients who underwent MRI of the cranium and orbit as part of work-up for vertigo and hearing impairment in the period from 2012 to 2022 were used as the initial dataset. The first 57 age- and gender matched patients without documented glaucoma, other optic neuropathies or intracranial diseases were included. Exclusion and inclusion criteria were verified in the medical records.

### MRI and CT acquisition

MRI -images were acquired with a 3 T whole body magnet (Skyra; Siemens Healthcare, Erlangen, Germany) with a 32-channel head coil using T2-weighted sequence using following parameters: TE/TR = 139/1400 ms, 0.7/0.9 mm slice thickness (tra/sag), flip angle = 120^o^, 1 average. CT – images were acquired after injection of 10 ml iopamidol (molecular weight 778 D, Iopamiro 300, Bracco, Milano, Italy) with a 64-detector scanner (Aquillion 64, Toshiba, Tokyo, Japan) providing 0.5 mm×32 section collimation. All subjects were instructed to look straight ahead during imaging.

### Optic nerve tortuosity measurements

**Fig. 1:**
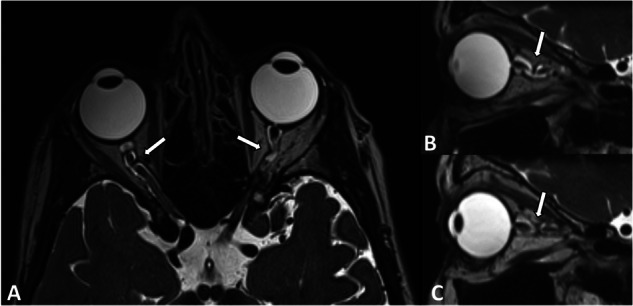
MRI images of a 67-year-old female normal tension glaucoma patient with optic nerve kinks in both planes in both optic nerves. **A** Kinking in the axial plane. **B** Kinking in the sagittal plane of the right optic nerve and **C** Kinking in the sagittal plane of the left optic nerve. Kinking marked with arrows.

**Fig. 2:**
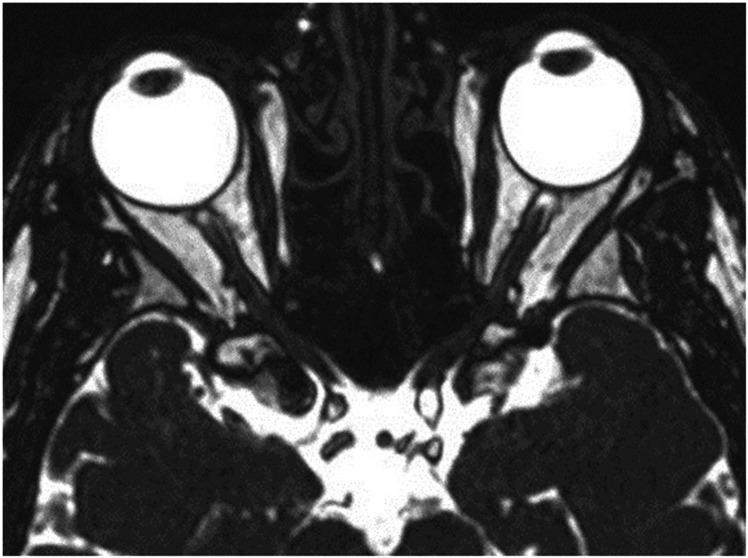
MRI images of a 71-year-old male control without kinking of the optic nerve. No kink visible in the axial plane.

Patients were divided in subgroups based on the occurrence of an optic nerve kink: 0 = straight or slightly curved optic nerve, 1 = visible kink of the optic nerve in axial images (1.1 = 1 kink, 1.2 = two kinks), 2 = visible kink of the optic nerve in sagittal images (2.1 = 1 kink, 2.2 = two kinks), 3 = visible kink of the optic nerve in both, axial and sagittal, images (Figs. 1 and 2). All radiological images were reviewed by two experienced neuro-radiologist and one experienced neuroophthalmologist blinded to the neuro-ophthalmological examination. Inter-rater agreement between the three readers on the presence of tortuosity was in 110/117 optic nerve, (94%).

### Statistical analysis

Statistical analysis was performed using SIGMAPLOT 10.0 (Systat Software, San Jose, CA, USA) and Microsoft Excel (Microsoft Corporation, Redmond, WA, USA). Data were analysed using Fisher’s two-tail exact test and the unpaired two-tailed t-test. For bivariate analysis the point-biserial’s correlation coefficient was used. All data are expressed as mean and standard deviation or median and interquartile ranges, respectively. Differences were considered significant when the error probability was *p* < 0.05.

## Results

Between 2012 and 2022 57 NTG patients (mean age 69 ± 10 years), 27 women (68 ± 8 years) and 30 men (70 ± 11 years) underwent imaging of the cranium and orbit (27 CTC and 30 MRI). 12 patients underwent both CTC and MRI. A total of 108 of 114 eyes of these patients (51 eyes in females and 57 eyes in males) fulfilled the inclusion criteria. Only one randomly selected (random generator function in Microsoft Excel) eye of each subject (57 eyes) was included in the statistical analysis. The mean glaucomatous visual field defect (MD) at time of CTT or MRI was 14 ± 7 dB (min. 4 dB, max. 27 dB) and the mean IOP was 14 ± 2 mmHg. The mean refractive error was −0.8 dioptres (D). None of the patients had a refractive error > +3 or < −3 dioptres (D).

The control group consisted of 57 age and gender matched controls (mean age 69 ± 9 years), 27 women (68 ± 9 years) and 30 men (70 ± 10 years) without known optic nerve diseases who underwent MRI of the cranium and orbit. Only one eye corresponding to the selected eye of the NTG group (51 eyes in females and 57 eyes in males) was included (Table 1).

**Table 1:** Number, Gender and Age of Normal Tension Glaucoma patients and controls.

Normal Tension Glaucoma			Controls		
	Number	Age in years [AM and SD]		Number	Age in years [AM and SD]
**All**	**57**	**69** ± **10**		**57**	**69** ± **9**
**Females**	**27**	**68** ± **8**		**27**	**68** ± **9**
**Males**	**30**	**70** ± **11**		**30**	**70** ± **10**

The number of normal tension glaucoma patients and controls was identical and there was no statistically significant age difference, neither in females nor in males. AM for arithmetic mean, SD for standard deviation.

**Fig. 3:**
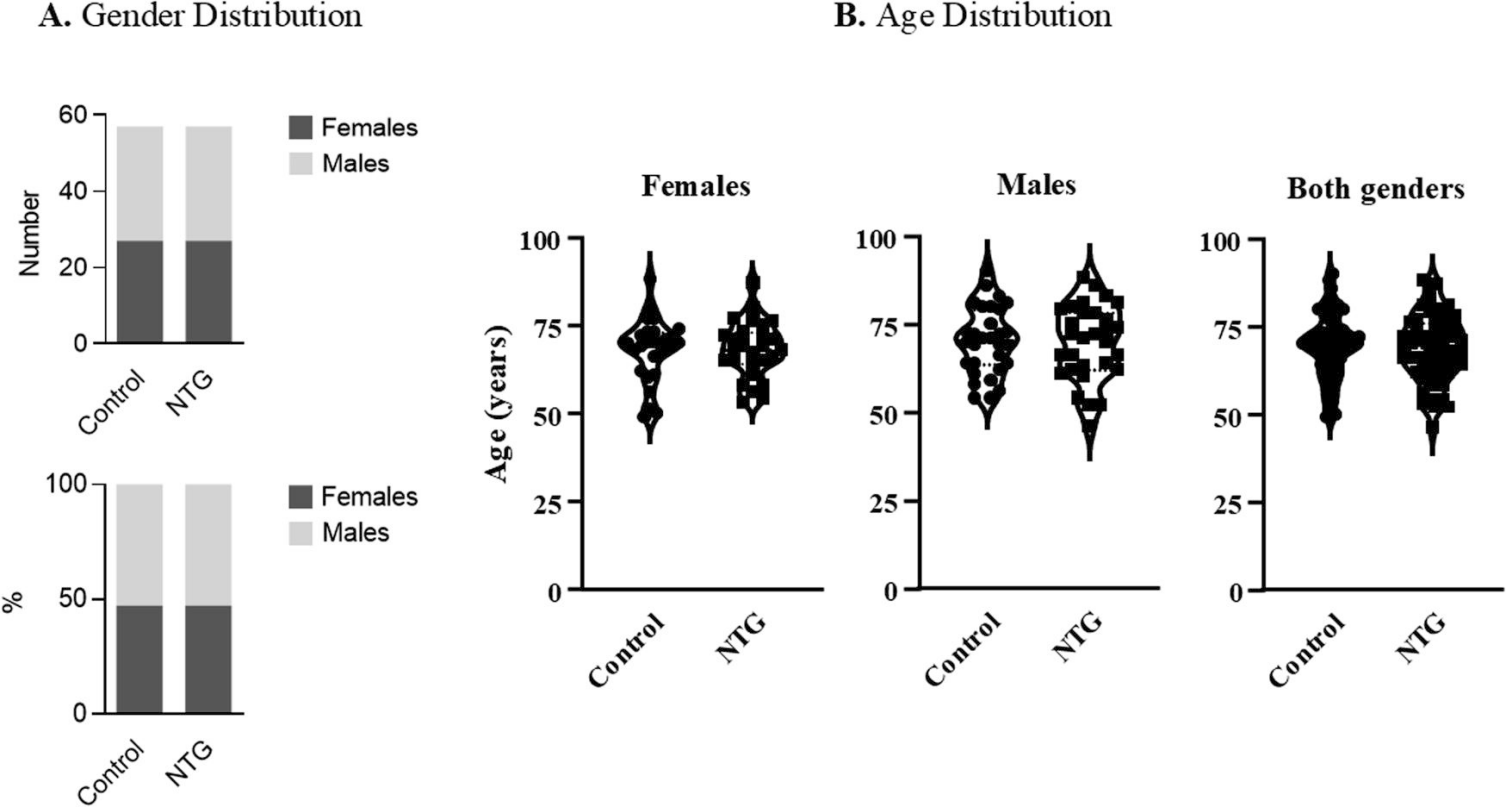
Gender and Age distribution in normal tension glaucoma and controls. **A** Gender Distribution. **B** Age Distribution.

The number of females and males was identical in the NTG group and the control group (Fig. 3A) and there was no statistically significant difference in age between the two groups, neither in females nor in males (females and males: *p* = 0.91, unpaired two-tailed t-test; females: *p* = 0.95, unpaired two-tailed t-test; males: *p* = 0.85, unpaired two-tailed t-test) (Fig. 3B).

### Incidence optic nerve kinking

**Fig. 4:**
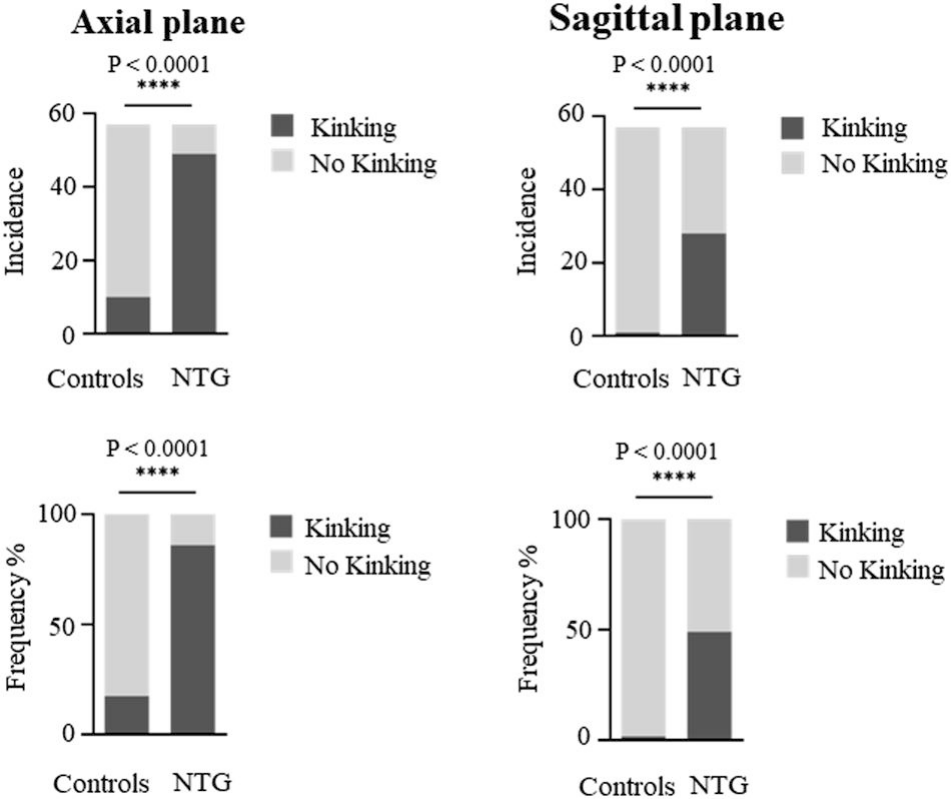
Incidence of optic nerve kinking in normal tension glaucoma and controls. Incidence in the (**A**) axial plane. Incidence in the (**B**) sagittal plane.

In the axial plane at least one optic nerve kink was found in 49 of 57 (86%) optic nerves in patients with NTG and in 10 of 57 (18%) optic nerves in controls while in the sagittal plane in 28 of 57 (49%) optic nerves in patients with NTG and in 1 of 57 (2%) optic nerves in controls. The difference between patients with NTG and controls showed statistical significance in both planes (axial plane: *p* < 0.0001; Fisher’s two-tailed exact test; sagittal plane: *p* < 0.0001; Fisher’s two-tailed exact test) (Fig. 4).

In all NTG patients where an optic nerve kink was found in the sagittal plane, an optic nerve kink was also found in the axial plane (28 of 57, 49%) while the subject in the control group who showed a vertical optic nerve kink did not have simultaneously an optic nerve kink also in the axial plane. In 3 of 57 (5%) optic nerves in NTG patients two kinks were found in the axial plane, while two kinks in the axial plane were not found in any of the control subjects.

There was no statistically significant difference of optic nerve kinking incidence between females and males neither in NTG patients nor in controls in the axial plane while a low statistically significant difference was found in the sagittal plane in NTG patients where an optic nerve kink was more common in females compared to males. (NTG: axial plane: *p* = 0.26, Fisher’s two-tailed exact test, sagittal plane: *p* = 0.02, Fisher’s two-tailed exact test; controls: axial plane: *p* = 0.73, Fisher’s two-tailed exact test, sagittal plane: *p* = 0.99, Fisher’s two-tailed exact test). In the 12 patients who underwent both CTC and MRI, the same kinking occurrence was found in both imaging modalities (CTC and MRI).

Bivariate analysis comparing the optic nerve kinking incidence with age showed no statistically significant correlation; nor was there a correlation in the axial nor in the sagittal plane in neither NTG patients (Point-Biserial’s correlation coefficient: axial plane r = −0.24, *p* = 0.08; sagittal plane r = 0.16, *p* = 0.23) nor in controls (Point-Biserial’s correlation coefficient: axial plane r = 0.23, *p* = 0.09; sagittal plane r = −019, *p* = 0.16).

## Discussion

This study demonstrates a statistically significant higher incidence of orbital optic nerve tortuosity in 57 Caucasian patients with NTG compared to 57 age- and gender-matched Caucasian control subjects without optic nerve diseases. The incidence of optic nerve tortuosity was significantly higher in both the axial (horizontal tortuosity) and sagittal (vertical tortuosity) planes.

Optic nerve tortuosity is a frequent finding in patients with elevated intracranial pressure, so it is likely that increased intracranial pressure is involved in the development of optic nerve tortuosity. But what about optic nerve tortuosity and glaucoma?

Demer et al. [17] were the first to study the optic nerve anatomy in 17 patients with NTG. They performed a prospective study with MRI and looked at the optic nerve configuration in central gaze, large angle adduction and abduction and found the optic nerve to be more redundant in central gaze and abduction in patients with NTG while they found no difference in adduction between healthy controls and NTG patients. A study by Özel et al. [18] using a tortuosity grading system (tortuosity index) to evaluate tortuosity in high tension primary open angle glaucoma (POAG), a cohort of ocular hypertension patients and a healthy control group found the highest tortuosity index in the POAG group and lowest in healthy controls. This is an interesting finding but limited to high tension POAG patients with no reference to NTG. These measurements are in striking contrast to a study [19] conducted in China on 10 high tension POAG subjects that found tauter optic nerves in their POAG group compared to healthy controls. The reason for this obvious discrepancy of the shape of the optic nerve in high tension POAG between these studies [17–19] is not clear but may be dependent on genetic variances between Caucasians and Asian ancestry.

All of the above studies defined optic nerve tortuosity as the deviation of the optic nerve length from the shortest distance (straight line) between two determined points of the optic nerve. While Demer et al. [17] and Wang et al. [19] have measured these deviations only on a part of the orbital optic nerve, Özel et al. [18] included the entire optic nerve from the eye globe to the optic chiasm. In contrast to these studies, we did not measure the optic nerve length but examined the orbital optic nerve for the occurrence of optic nerve kinks. It would be interesting to know whether such optic nerve kinks were also found in the studies by Demer et al. [17] and Özel et al. [18] and whether such large kinks caused the greater optic nerve redundancy in primary gaze in these studies.

In the current study optic nerve kinks were found in both examined planes, in the axial plane, referred as horizontal tortuosity and in the sagittal plan, referred as vertical tortuosity. Horizontal tortuosity was found in 49 of 57 (86%) optic nerves in patients with NTG and in 10 of 57 (18%) optic nerves in controls while vertical tortuosity in 28 of 57 (49%) optic nerves in patients with NTG and in 1 of 57 (2%) optic nerves in controls. Brodsky et al. [12] confined in a retrospective case series on MRI findings in pseudotumor cerebri their tortuosity analysis to the vertical component because they argued that some tortuosity may exist in normal subjects. Further, the ability of axial MR imaging to display relatively minor degrees of horizontal tortuosity makes it a relatively nonspecific finding. In accordance with this assumption, horizontal tortuosity was indeed found in about 18% of the healthy control group, but in 86% of the NTG group. In accordance with this assumption horizontal tortuosity was indeed found in about 18% also in the healthy control group, in the NTG group however in 86%. In the study by Brodsky et al. [12] vertical tortuosity was noted in 8 of 20 (40%) of patients with pseudotumor cerebri with high intracranial pressure while only in 1 of the 20 (5%) healthy controls. In the current study the incidence of vertical tortuosity was even higher (28 of 57, 49%) in NTG patients while in the control group the incidence was slightly lower (1 of 57, 2%) compared to the findings published by Brodsky et al. [12].

The mechanisms that might lead to a higher incidence of optic nerve kinking in patients with NTG are not known and can only be speculated. The kinking of the optic nerve could be considered analogous to buckling in blood vessels, [20] where venous buckling might be considered analogous to optic nerve kinking. Biomechanical modelling of this phenomenon predicts there is a critical pressure at which venous buckling occurs with high pressure or reduced axial strain generating increased risk [21]. Greater redundancy in the optic nerve would lead to reduced axial strain, which has been reported to a greater degree with NTG. [17] As the intracranial pressure in patients with NTG is normal [22] or according to some studies [23] even abnormally low, a generalized high intracranial pressure might not play a role in the development of optic nerve kinking in patients with NTG. Enlarged optic nerve sheath diameters, similar to those in patients with idiopathic intracranial hypertension (IIH) have been measured also in patients with NTG, [24] which are suggestive of locally elevated CSF pressure in the subarachnoid space of the orbital optic nerve. Such enlarged optic nerve sheath diameters have been noted also in many of the NTG patients included in this study. One reason for a locally elevated pressure in the SAS behind the lamina cribrosa could be a compartmentation of the orbital optic nerve subarachnoid space. Whether optic nerve kinking is involved in the development of elevated CSF pressure, or the effect of a higher pressure needs to be addressed in further studies. Optic nerve kinking, however, involves a narrowing of the area of the subarachnoid space and therefore likely influences the CSF flow dynamics along the orbital subarachnoid space [25]. Its possible role involved in the pathophysiology of NTG needs to be evaluated.

This study has several limitations. First, the retrospective design of the study that includes two different imaging modalities in the NTG group, CTC (*n* = 27) and MRI (*n* = 30), while in the control group only MRI was performed. However, the imaging technique (MRI or CTC) is unlikely to have an influence on the detection of optic nerve tortuosity. Both imaging modalities (CTC and MRI) were performed in 12 NTG patients and no differences were found. Second, we did not measure optic nerve kinking in add- and abduction and can therefore not provide any information about kinking in different globe positions. Third, the included control subjects did not undergo an ophthalmological examination. Glaucoma and other optic nerve diseases were excluded based on the medical records. Fourth, all NTG patients were in an advanced stage of the disease. Whether severe axon loss influences the development of optic nerve tortuosity is not known and needs to be examined in further studies.

In summary this study demonstrates a high statistically significant incidence of optic nerve kinking in patients with NTG compared to controls without known optic nerve diseases. The altered anatomy can be expected to influence the CSF flow dynamics within the orbital optic nerve subarachnoid space. A possible connection to the pathophysiology of NTG needs to be further evaluated. Based on the data from this study we suggest MRI studies of the orbit in NTG patients with progressive visual field loss.

## Summary

### What was known before

- The role of cerebrospinal fluid dynamics along the optic nerve and the link to neurodegenerative diseases such as Alzheimer’s has recently gained interest for the understanding of the pathophysiology of glaucoma, particularly normal tension glaucoma. The majority of studies examine the optic disc in these patients. There are however only a few studies that deal with the entire optic nerve.

### What this study adds

- This study investigated the anatomy of the orbital optic nerves on radiological images in normal tension glaucoma patients and age- and gender-matched controls without known optic nerve diseases. This study found a significantly higher incidence of optic nerve kinking in patient with normal tension glaucoma compared to controls. This finding suggest that the altered anatomy might influence the cerebrospinal fluid dynamics within the subarachnoid space as well as the intraparenchymal flow of interstitial fluid.

## Data Availability

Data of this study are available upon reasonable request to the corresponding author.
